# Variability in human attractiveness to mosquitoes

**DOI:** 10.1016/j.crpvbd.2021.100058

**Published:** 2021-11-02

**Authors:** Joel Henrique Ellwanger, Jáder da Cruz Cardoso, José Artur Bogo Chies

**Affiliations:** a Laboratório de Imunobiologia e Imunogenética, Programa de Pós-Graduação em Genética e Biologia Molecular - PPGBM, Departamento de Genética, Universidade Federal do Rio Grande do Sul - UFRGS, Porto Alegre, Rio Grande do Sul, Brazil; b Divisão de Vigilância Ambiental em Saúde, Centro Estadual de Vigilância em Saúde, Secretaria da Saúde do Estado do Rio Grande do Sul, Porto Alegre, Rio Grande do Sul, Brazil

**Keywords:** Culicidae, Genetics, Human attractiveness, Microbiota, Mosquito, Mosquito bites

## Abstract

Blood-feeding mosquitoes locate humans spatially by detecting a combination of human-derived chemical signals, including carbon dioxide, lactic acid, and other volatile organic compounds. Mosquitoes use these signals to differentiate humans from other animals. Spatial abiotic factors (e.g. humidity, heat) are also used by mosquitoes to find a host. Mosquitoes cause discomfort and harm to humans, being vectors of many pathogens. However, not all humans suffer from mosquito bites with the same frequency or intensity. Some individuals are more attractive to mosquitoes than others, and this has an important impact on the risk of infection by pathogens transmitted by these vectors, such as arboviruses and malaria parasites. Variability in human attractiveness to mosquitoes is partially due to individual characteristics in the composition and intensity in the release of mosquito attractants. The factors that determine these particularities are diverse, modestly understood and still quite controversial. Thus, this review discusses the role of pregnancy, infection with malaria parasites (*Plasmodium* spp.), skin microbiota, diet, and genetics in human attractiveness to mosquitoes. In brief, pregnancy and *Plasmodium* infection increase the host attractiveness to mosquitoes. Skin microbiota and human genetics (especially HLA alleles) modulate the production of mosquito attractants and therefore influence individual susceptibility to these insects. There is evidence pointing to a role of diet on human susceptibility to mosquitoes, with some dietary components having a bigger influence than others. In the last part of the review, other factors affecting human-mosquito interactions are debated, with a special focus on the role of mosquito genetics, pathogens and environmental factors (e.g. wind, environmental disturbances). This work highlights that individual susceptibility to mosquitoes is composed of interactions of different human-associated components, environmental factors, and mosquito characteristics. Understanding the importance of these factors, and how they interact with each other, is essential for the development of better mosquito control strategies and studies focused on infectious disease dynamics.

## Highlights

• Individual human attractiveness to mosquitoes is highly variable.
• Mosquito attractants released into the air vary from person to person.
• Variation in attractiveness to mosquitoes alters the risk of mosquito-borne infections.
• Pregnancy, malaria infection, skin microbiota and genetic factors alter the release of mosquito attractants.
• Environment and mosquito-related factors affect human–mosquito interactions.

## Graphical abstract

**Figure:**
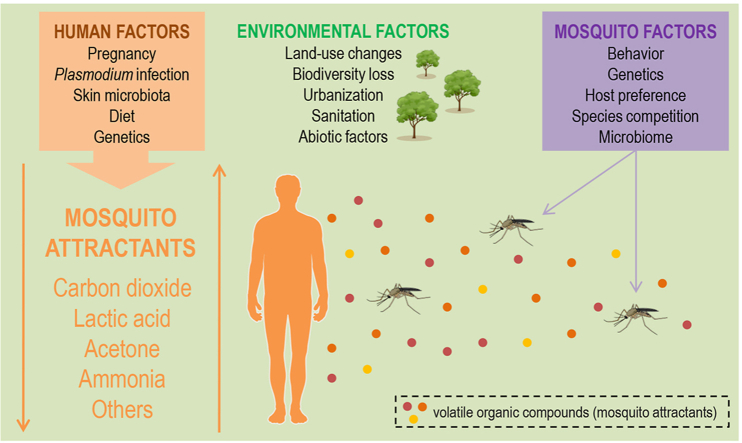

## 1 Introduction

Mosquitoes (Diptera: Culicidae) cause huge social and medical damage to humans since these insects are major contributors to the burden of vector-borne infections worldwide. Mosquitoes of different species are vectors of diseases such as dengue, malaria, congenital Zika syndrome, lymphatic filariasis, yellow fever, West Nile fever, chikungunya, among many others. *Plasmodium* alone causes 212 million human malaria cases annually, mainly in tropical countries (Franklinos et al., 2019). In addition to the damage caused by diseases transmitted by mosquitoes, these insects are responsible for a series of non-lethal impacts to the daily lives of individuals living in mosquito-infested areas due to the bites that cause pain, allergic reactions, besides other physical and psychological discomforts, which disrupt people’s work activities and sleep quality.

Although the problems mentioned above are common to different populations worldwide, not all individuals suffer from the problems caused by mosquitoes to the same degree, not only due to different geographical distribution of mosquitoes, but also because humans are not equally attractive to these insects. For instance, in a given environment, while some individuals suffer from a large number of bites, others are little bothered by mosquitoes (Enserink, 2002). Indeed, multiple experimental approaches have shown that human attractiveness to mosquitoes is variable between different individuals (Schreck et al., 1990; Lindsay et al., 1993; Knols et al., 1995; Qiu et al., 2006; Verhulst et al., 2011; Omolo et al., 2013).

Heat and volatile organic compounds released into the air by humans are signals used by female mosquitoes to locate humans and then to obtain a blood meal. However, these signals are not homogeneously released by humans. There is great variability in the release of mosquito attractants between individuals. This variability explains, at least in part, why some individuals are preferred by mosquitoes (Enserink, 2002; Smallegange et al., 2011). There are also data indicating that the particular attractiveness of an individual to mosquitoes is stable over time (Lindsay et al., 1993; Qiu et al., 2006). This information reinforces the fact that each individual has characteristics concerning attractiveness to mosquitoes and susceptibility to bites.

An often-neglected factor, not mentioned in many studies addressing human-mosquito interactions, differences in individual attractiveness to mosquitoes modify the risk of infectious diseases transmitted by these insects (Lindsay et al., 1993). Of note, epidemiological modeling studies should take into account that not all individuals are equally susceptible to mosquito bites. In addition, individual particularities regarding mosquito attractiveness can influence the results from studies carried out with human traps, eventually even hindering comparisons between such studies (Knols et al., 1995). Ultimately, identifying mosquito-attracting molecules can help the development of better mosquito traps and repellents, as well as novel tools for mosquito capture and control (Enserink, 2002). For example, the identification of highly efficient mosquito attractants could be applied in better “mosquito magnet” devices for home use and in a new generation of traps to be used in scientific research. Moreover, individuals with high susceptibility to mosquito bites (potentially identified through genetic or chemical testing) could be advised to take additional precautionary measures against mosquitoes (use of repellents, long clothing, bednets) when visiting endemic areas for mosquito-borne diseases. Moreover, the study and manipulation of skin microorganisms and associated mosquito attractants to create microbial-based repellants, thus reducing the human attractiveness to mosquitoes, is on the frontier of vector control strategies (Lucas-Barbosa et al., 2021).

Although it is well established that human attractiveness to mosquitoes is mainly regulated by the release of volatile organic compounds, the factors that influence the pattern of release of these signals (considering composition and intensity) and that, as a consequence, affect individual susceptibility to mosquitoes, are not fully understood. For instance, the influence of some factors, such as human genetic traits (Kirk et al., 2000; Verhulst et al., 2013; Fernández-Grandon et al., 2015; Jones et al., 2017), has only recently been demonstrated. The role of other factors, such as diet, is still controversial. Therefore, the purpose of this review is to describe and discuss the role of human factors in attractiveness to mosquitoes, as well as adding to this discussion the impact of mosquito genetics, mosquito-associated pathogens and environmental factors (wind patterns, anthropogenic changes in the landscape, among others) on human-mosquito interactions and variability in human attractiveness to mosquitoes. Of note, the influence of skin microbiota, genetics, and infection by parasites on differential attraction in mosquito-human interactions was recently reviewed by Martinez et al. (2021), with a special focus on the evolutionary aspects involved in such interactions. These authors also discussed how understanding these interactions can contribute to the development of better disease control tools (Martinez et al., 2021). Other important works addressed specifically the impact of skin microbiota on attractiveness to arthropod vectors and how the study of skin bacterial volatiles can help in the development of better vector control strategies, see e.g. Verhulst et al. (2018a) and Lucas-Barbosa et al. (2021). Our review expands this discussion by exploring other under-discussed factors (e.g. diet) within the One Health perspective, in which human, animal and environmental factors are taken into account to understand and mitigate zoonotic and vector-borne diseases. In this sense, our article addresses the role of non-human factors in human-mosquito interactions, including wind, mosquito behavior and microbiome, among others. Before discussing these aspects, this article presents a brief review of the basic aspects concerning human-mosquito interactions. In the last part of the article, other factors affecting human-mosquito interactions are discussed, with a special focus on the role of mosquito genetics, mosquito-associated pathogens, and environment-related factors.

## 2 Basic aspects of human-mosquito interactions and mosquito attractants

The attractiveness of humans to mosquitoes can be understood as based on two basic steps. The first step is the attraction of the mosquito from long distances to the proximity of an individual. The second step is the mosquito bite *per se*. It is interesting to note that a greater attractiveness does not necessarily result in more bites, which may depend on factors such as the defensive action of the individual in relation to the presence of mosquitoes (Lindsay et al., 1993).

Mosquitoes use humidity, heat, and visual and olfactory stimuli to guide the flight, landing, and to find a food source (McMeniman et al., 2014; Ray, 2015; Raji & DeGennaro, 2017). Carbon dioxide (CO_2_), lactic acid, acetone, and ammonia are some of the most well-known volatile human-derived signals used by mosquitoes to locate humans (Smallegange et al., 2011; Takken & Verhulst, 2013; Raji & DeGennaro, 2017; Dormont et al., 2021). Anthropophilic mosquitoes differentiate humans from other animals (e.g. cows) by detecting volatile organic compounds emitted characteristically by humans, although some mosquito species cannot significantly differentiate human odors from non-human primate odors (Smallegange et al., 2011; Takken & Verhulst, 2013; Raji & DeGennaro, 2017; Verhulst et al., 2018b). These human-derived signals are called “kairomones”, which are substances, or chemical signals, that mediate the interaction between different species, benefiting only the species that receives the chemical signal (Sbarbati & Osculati, 2006), in the present case, mosquitoes. In this article, the terms “kairomones” and “mosquito attractants” will be used interchangeably.

Kairomones are interpreted by mosquitoes in combination as compound blends. In this sense, the attraction of *Anopheles gambiae* (*sensu stricto*) and *Aedes aegypti* mosquitoes to a human host is mediated by the synergic effect of CO_2_, lactic acid, and other volatile components. Conversely, a kairomone alone, such as lactic acid or CO_2_, may have a reduced effect as a mosquito attractant compared to a combination of human-derived kairomones (Geier & Boeckh, 1999; Dekker et al., 2002).

Kairomones are detected by mosquitoes through sensory (olfactory) receptors found in the antennae, labellum and maxillary palps. These receptors include odorant, gustatory, and ionotropic receptors (Takken & Verhulst, 2013; Ray, 2015; Raji & DeGennaro, 2017). Mosquitoes detect CO_2_ through the gustatory receptors, composed of Gr1, Gr2 and Gr3 sub-units found in maxillary palps, with a predominant role played by the sub-units Gr1 and Gr3 (Erdelyan et al., 2012; McMeniman et al., 2014). CO_2_ induces flight takeoff and also sustains the flight, being the main molecule used by mosquitoes to find a blood source over long distance (Ray, 2015; Raji & DeGennaro, 2017). There is evidence showing that in female *Ae. aegypti* the synergic detection of CO_2_, heath and other human-derived kairomones is mediated especially by the Gr3 sub-unit (McMeniman et al., 2014). Despite the specificities of distinct mosquito sensory receptors, the detection system of volatile kairomones is likely robust and redundant (Raji & DeGennaro, 2017).

According to Dekker et al. (2002), humans release l-lactic acid (L-la) from skin in much larger quantities (mean L-la: 151 μg/ml) than other vertebrates, such as *Pan troglodytes* (chimpanzee; mean L-la: 13.5 μg/ml), *Macaca mulatta* (Rhesus monkey; mean L-la: 9.7 μg/ml), *Sus scrofa* (pig; mean L-la: 6.8 μg/ml), *Bos taurus* (cow; mean L-la: 9.4 μg/ml), *Capra hircus* (goat; mean L-la: 2.5 μg/ml), *Ovis aries* (sheep; mean L-la: 9.7 μg/ml), *Equus caballus* (horse; mean L-la: 3.3 μg/ml), *Llama glama* (llama; mean L-la: 5.6 μg/ml), *Canis familiaris* (dog; mean L-la: 5.7 μg/ml), *Felis cattus* (cat; no L-la detected), *Rattus norvegicus* (rat; no L-la detected), *Oryctolagus cuniculus* (rabbit; no L-la detected), and *Gallus* *gallus* (chicken; L-la < 1.0 μg/ml). This indicates that lactic acid could be considered a mosquito attractant characteristic of humans (Dekker et al., 2002). However, it cannot be ruled out that each anthropophilic mosquito species uses different kairomones to specifically recognize humans. Bernier et al. (2000) detected more than 300 volatile human-derived substances, of which more than 270 were considered potential *Ae. aegypti* attractants. Recently, Dormont et al. (2021) listed 105 proven attractants of one or more mosquito species, including 77 volatile compounds. For example, sulcatone (6-methyl-5-hepten-2-one), an odorant found in elevated quantities in humans as compared to other vertebrates, seems to be a major component for the recognition of human odors by *Ae. aegypti*, being recognized by the odorant receptor Or4. In this sense, sulcatone can be considered a human-specific odorant (McBride et al., 2014). However, the effect of sulcatone on mosquitoes is complex since this compound can act as a mosquito attractant or a repellent, depending on the dose and combination with other compounds (Logan et al., 2008, 2010; McBride et al., 2014; Menger et al., 2014). Thus, humans indeed produce skin-derived volatile compounds associated with mosquito unattractiveness; some of them (e.g. geranylacetone, sulcatone at high concentrations) potentially acting as natural repellents. Also, such compounds can act through a “masking effect”, in this case interfering in the interaction of mosquitoes with other human-derived common signals (Costantini et al., 2001; Logan et al., 2010; Menger et al., 2014). However, the actual effects of these repellents on the behavior of mosquitoes in the natural environment are still little explored.

In summary, the information presented above indicates that both the intensity and the combination of human-derived mosquito attractants released by a given individual influence the susceptibility to mosquitoes. The next section of this review will detail the factors that influence these aspects.

## 3 Variability in the production of mosquito attractants by humans

Different human individuals generally produce similar types of volatile compounds. However, the amount of compounds emanating from human skin varies among subjects (Bernier et al., 2000). For instance, the production of lactic acid by humans is quite variable. Particularities in eccrine gland density, metabolic rate and differential skin pH can explain variations in the lactic acid production between different individuals (Dekker et al., 2002; Smallegange et al., 2011). Also, the rates of CO_2_ release from each person will depend on factors such as metabolic rate, body mass and respiratory activity. The release of ammonia and other carboxylic acids will also vary according to metabolic rates and other individual characteristics. This information is relevant because it indicates that the release rates of CO_2_, ammonia and other volatile compounds by humans are variable and circumstantial. However, it is essential to consider that such variations caused by respiratory rate or other metabolic circumstances will not necessarily have a significant impact on the attractiveness to mosquitoes. This will depend on the intensity of the variation, distance between humans and mosquitoes, atmospheric and environmental conditions interfering with the dispersion of mosquito attractants, among other factors. Moreover, considering that many volatile components are present in human sweat, glandular alterations and skin microbial compositions will have an important impact on the release rates of mosquito attractants (Smallegange et al., 2011). Therefore, particularities in the production of generalist kairomones such as CO_2_, lactic acid and ammonia could be associated with differences in susceptibility to mosquitoes.

Alternatively, some authors suggest that it is unlikely that the variation in attractiveness to mosquitoes is due to differential production of CO_2_ or lactic acid precisely because of their generalist characteristics. The variations in human attractiveness to mosquitoes would be attributed to secondary volatile compounds released directly by the skin or metabolized by the skin microbiota (Knols et al., 1995; Logan et al., 2008). The variable combination or particular amount of these secondary molecules produced by different individuals could better explain the differences in attractiveness to mosquitoes (Logan et al., 2008; Smallegange et al., 2011). For example, as previously mentioned, individuals who release lower doses of sulcatone would be more attractive to mosquitoes (McBride et al., 2014). In addition, considering the large number of volatile compounds produced by humans (over 300, as shown by Bernier et al. (2000)), it makes sense that differences in the combination of different secondary compounds released by each individual have a major impact on susceptibility to mosquitoes.

**Fig. 1:**
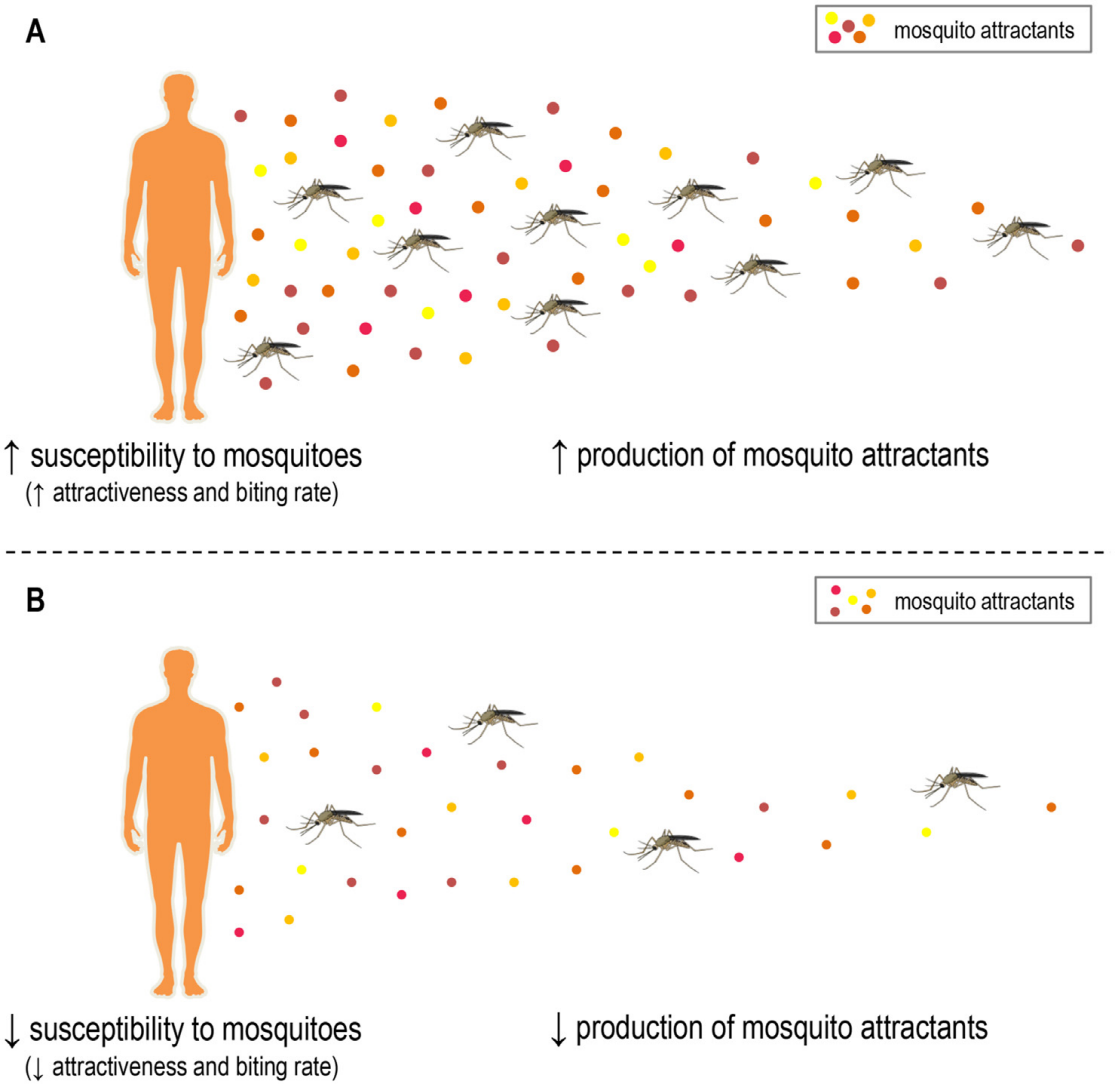
Differences in human susceptibility to mosquitoes due to the intensity in the production of mosquito attractants. Individuals who produce mosquito attractants (kairomones) more intensely may show increased susceptibility to mosquitoes, considering attractiveness and biting rate (**A**). Alternatively, individuals who produce a reduced amount of mosquito attractants may show reduced susceptibility to mosquitoes (**B**). Mosquito attractants (e.g. CO_2_, lactic acid, ketones, ammonia) are represented by colored dots. The gradual molecular diffusion of volatile compounds is a visual simplification. It is necessary to consider that host-derived volatile kairomones can be dispersed into the air as “packages” and depending on the wind direction. See the main text for references. Human illustrations were obtained from *Servier Medical Art* (available at https://smart.servier.com, under a Creative Commons Attribution 3.0 Unported License). Mosquito illustrations were obtained from *Mind the Graph* (available at www.mindthegraph.com).

**Fig. 2:**
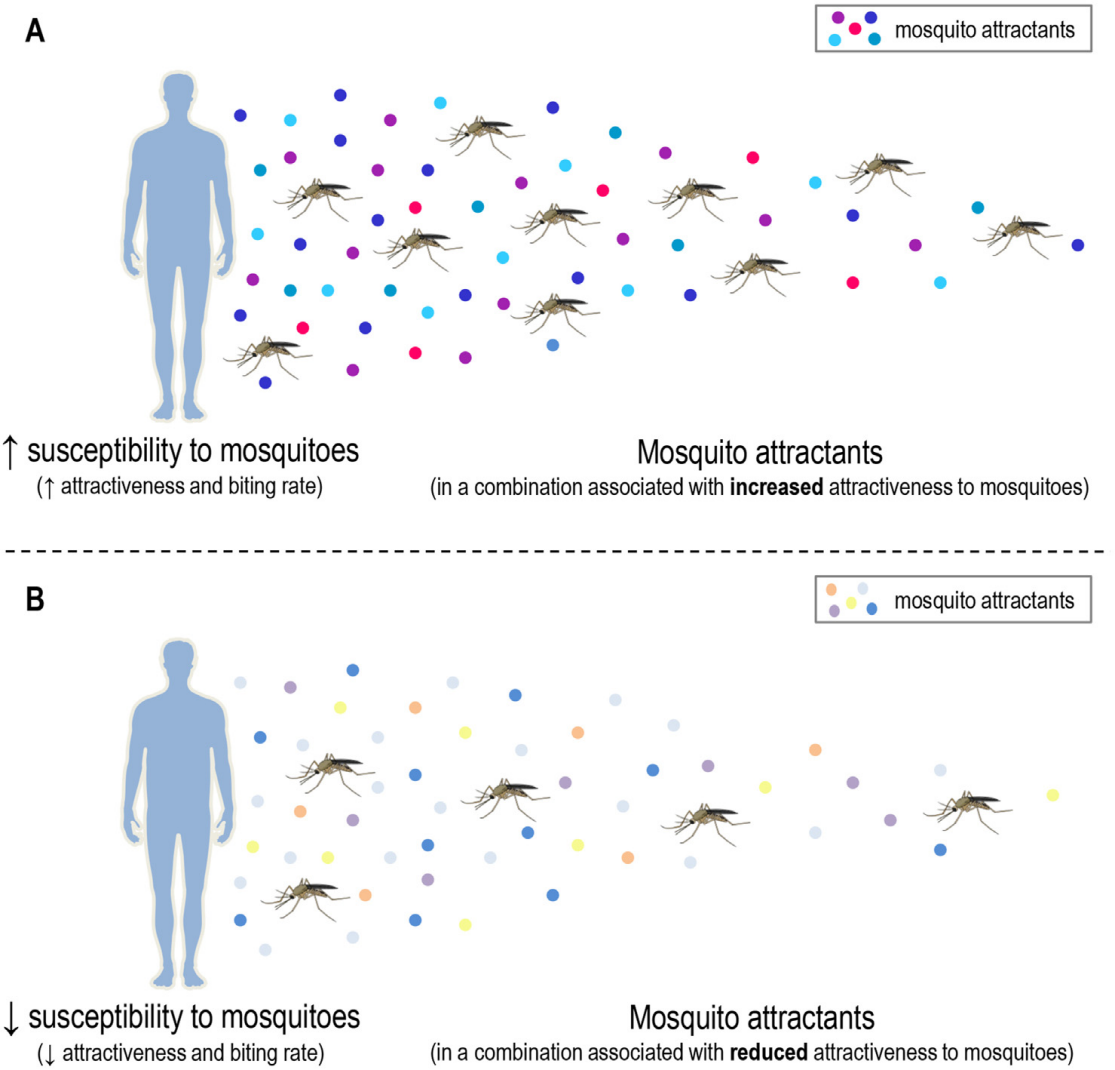
Differences in susceptibility to mosquitoes due to differences in the combination (blend) of mosquito attractants. Increased susceptibility to mosquitoes may be due to the production of a combination of mosquito attractants (kairomones) associated with greater mosquito attraction, considering attractiveness and biting rate (**A**). On the other hand, reduced susceptibility to mosquito attraction and biting rate may be due to the production of a combination of mosquito attractants associated with reduced mosquito attraction. Of note, the production of natural mosquito repellents can also contribute to a reduced human susceptibility to mosquitoes (**B**). Mosquito attractants are represented by colored dots. The gradual molecular diffusion of volatile compounds is a visual simplification. It is necessary to consider that host-derived volatile compounds can be dispersed into the air as “packages” and depending on the wind direction. See the main text for references. Human illustrations were obtained from *Servier Medical Art* (available at https://smart.servier.com, under a Creative Commons Attribution 3.0 Unported License). Mosquito illustrations were obtained from *Mind the Graph* (available at www.mindthegraph.com).

Finally, an integrative explanation is also possible. Considering that mosquitoes use a combination of “generalist” and “secondary” volatile compounds to guide the flight and find a food source, it is plausible that variations in the emanation of the two types of kairomones have an impact on the individual susceptibility to mosquitoes. In this sense, both variations in the amount (Fig. 1) and in the combination (Fig. 2) of generalist and secondary kairomones are important. Although the occurrence of such variations is well established, the factors and physiological circumstances underlying these particularities still need to be better understood. The next sections of this article will focus on the role of pregnancy, *Plasmodium* infection, skin microbiota, diet, and genetics in the production of volatile human-derived mosquito attractants.

### 3.1 Pregnancy

Different studies report that pregnant women show increased attractiveness to *An. gambiae* (*sensu lato*) (Lindsay et al., 2000; Ansell et al., 2002), *An. arabiensis* (Himeidan et al., 2004), and *Mansonia* spp. mosquitoes (Lindsay et al., 2000). This increase in attractiveness results, therefore, in increased susceptibility to mosquito-borne diseases, including malaria. More than a nuisance during pregnancy, this phenomenon is worrying since gestational malaria is a very serious condition associated with low birth weight, preterm delivery, stillbirth, as well as maternal morbidity and mortality (Desai et al., 2007).

Behaviors associated with increased exposure of pregnant women to mosquitoes (e.g. because they come out from under mosquito nets more often to urinate at night) may help explain the greater number of mosquito bites suffered by pregnant women compared to non-pregnant women. However, other biological factors also explain this phenomenon. Pregnant women probably release greater amounts of mosquito attractants (e.g. CO_2_) due to the higher metabolic rate and heat production observed during pregnancy (Lindsay et al., 2000; Ansell et al., 2002). From a broader perspective, this reinforces the fact that human attractiveness to mosquitoes, in addition to varying between different individuals, can be modified depending on the physiological and metabolic conditions observed in a particular individual.

As mentioned above, evidence suggests that pregnancy induces the release of a greater amount of classical mosquito attractants, especially CO_2_. However, it cannot be ruled out that pregnancy induces the release of some volatile organic compounds specifically associated with the gestational period (pregnancy-related odor signature) that end up acting as mosquito attractants (Lindsay et al., 2000). However, this assumption still needs to be investigated experimentally.

Most of the studies evaluating the impact of pregnancy on susceptibility to mosquitoes were carried out with mosquito species of the subfamily Anophelinae (Lindsay et al., 2000; Ansell et al., 2002; Himeidan et al., 2004). It is not yet clear whether pregnant women are also more attractive to non-anopheline mosquitoes. This information is highly relevant due to the epidemiological importance of the pathogens carried by species of the subfamily Culicinae, including dengue and Zika viruses. For example, if pregnant women are also more attractive to *Ae. aegypti*, this can have serious implications for the risk of diseases such as congenital Zika syndrome. Although this phenomenon is quite plausible, the little evidence available regarding this aspect does not indicate a statistically significant effect of pregnancy on the attractiveness of *Ae. aegypti* mosquitoes or other culicines (Lindsay et al., 2000; Himeidan et al., 2004). Only a borderline effect has been observed in the study performed by Lindsay et al. (2000).

### 3.2 *Plasmodium* infection

The chances of an individual being bitten by mosquitoes can increase when they suffer from malaria. There is strong evidence that humans infected with the transmissible gametocyte stage of malaria parasites (e.g. *Plasmodium falciparum*) show increased attractiveness to *An. gambiae* (*s.s.*) mosquitoes. This indicates that the parasite, specifically during the gametocyte stage, manipulates the host physiology (e.g. breath, body odors) and, consequently, the vector behavior, increasing the chances of transmission of the parasite to mosquitoes (Lacroix et al., 2005). These results were supported by other studies in humans [Batista et al. (2014), considering *An. darlingi* and *Plasmodium vivax*; Busula et al. (2017a), considering *An. gambiae* (*s.s.*) and *P. falciparum*] and a rodent model (De Moraes et al., 2014). Of note, De Moraes et al. (2014) reported increased attraction of *An. stephensi* mosquitoes to odors from *P. chabaudi*-infected mice compared to control mice.

Humans with symptomatic malaria infection produce volatile compounds in a characteristic way, which can differentiate them from uninfected individuals and even from individuals with asymptomatic malaria (De Moraes et al., 2018). In other words, malaria infection affects the release of kairomones, producing an “odor signature” of the host (de Boer et al., 2017; De Moraes et al., 2018), also known as “malaria smell” (Batista et al., 2014). This odor signature has the potential to be used in high sensitivity diagnostic tests, enabling infection detection in both symptomatic and asymptomatic stages (De Moraes et al., 2018). Data obtained by Robinson et al. (2018) showed that *Plasmodium* infection increases the attractiveness of children’s skin odor to mosquitoes, an effect attributed to the release of the aldehydes heptanal, octanal, nonanal, (*E*)-2-octenal and (*E*)-2-decenal, which are produced in greater amounts by infected subjects (Robinson et al., 2018). Also, Berna et al. (2015) found that thioethers are other components of malaria-associated smell, based on the analysis of breath specimens from *Plasmodium*-infected individuals. Taken together, these data indicate that there are mosquito attractants released specifically during *Plasmodium* infection, consistent with the existence of a “malaria smell”. However, it is still necessary to establish whether, or to what extent, the data obtained under experimental conditions represent what occurs under natural conditions (Stanczyk et al., 2017). Therefore, data reporting the effects of *Plasmodium* infection on host attractiveness to mosquito vectors are consistent, but still should be further investigated in the field.

It is believed that malaria-induced attractiveness can occur through the manipulation of the host’s odors release system or directly through *Plasmodium*-released odorants (Busula et al., 2017a). Interestingly, *P. falciparum* influences *An. gambiae* (*s.l.*) blood-meal seeking and feeding behaviors and also increases vector susceptibility to infection through the production of an isoprenoid precursor [(*E*)-4-hydroxy-3-methyl-but-2-enyl pyrophosphate (HMBPP)] (Emami et al., 2017). A clear sequence of events can explain this effect. In brief, HMBPP induces red blood cells to release CO_2_ and other volatile compounds in experimental conditions, suggesting that the *Plasmodium*-induced HMBPP production could increase human attractiveness to mosquitoes (Emami et al., 2017). To the best of our knowledge, this is the more consistent evidence on how malaria infection can increase the human attractiveness to mosquitoes. More recently, Debebe et al. (2020) showed that HMBPP-induced volatiles indeed can attract *Anopheles* mosquitoes under field conditions. Taken together, this evidence suggests that HMBPP would be an inducer of mosquito attractants, not an attractive molecule *per se*. This would be the most plausible explanation since in natural conditions mosquitoes do not choose the host based on molecules circulating in host’s blood, but rather through the identification of attractants released into the air. In this sense, how HMBPP-related production of mosquito attractants in the blood is translated into the release of these molecules into the air remains to be further elucidated.

Interestingly, malaria appears to cause changes not only in infected humans but also in mosquitoes. In this sense, *An. gambiae* (*s.s.*) female mosquitoes infected with *P. falciparum* sporozoites are more attracted to human kairomones compared to uninfected mosquitoes (Smallegange et al., 2013). In brief, alterations observed in *Plasmodium*-infected mosquitoes and humans that facilitate the transmission of the parasite are well supported by the literature (Lacroix et al., 2005; Smallegange et al., 2013; Batista et al., 2014; Busula et al., 2017a; Stanczyk et al., 2017). It is quite common to interpret that the malaria parasite “manipulates” the hosts in order to increase its transmission. However, many of these alterations may be consequences of the parasite’s effects on the immune system of mosquitoes and humans, but not directly due to a manipulation of the host by the parasite (Stanczyk et al., 2017). Regardless of how this phenomenon is interpreted, from an evolutionary perspective, the changes caused by *Plasmodium* on hosts appear to be advantageous for the parasite’s transmission, and probably for this reason the phenomenon has been selected and preserved. Beyond that, the effects caused by malaria on human susceptibility to mosquitoes reinforce that the release of mosquito attractants varies as a result of specific physiological situations.

Finally, as discussed elsewhere in this article, there are transient changes in human attractiveness to mosquitoes due to specific circumstances (e.g. pregnancy, *Plasmodium* infection) and stable long-lasting differences between people in the absence of transient circumstances. Therefore, it is essential to consider that variability in human attractiveness to mosquitoes observed during malaria infection may not necessarily or exclusively be due to the active host manipulation by *Plasmodium* parasites, especially considering that malaria induces several metabolic changes during disease (fever, sweating, among others) that could interfere with human-mosquito interactions. Also, baseline attractiveness to mosquitoes is difficult and complex to measure experimentally, and these issues must be taken into account when interpreting studies involving the effects of *Plasmodium* infection on human attractiveness to mosquitoes.

### 3.3 Skin microbiota

The skin bacteria *Bacillus cereus*, *Bacillus subtilis*, *Brevibacterium epidermidis*, *Corynebacterium minutissimum* and *Staphylococcus epidermidis* produce many mosquito attractants, including butyl acetate, butyl 2-methylbutanoate, butyl isobutyrate, dimethyldisulfide, 3-methyl-1-butanol, and 3-methylbutanoic acid (Verhulst et al., 2010a; Takken & Verhulst, 2017). Skin microbiota influences the release of mosquito attractants because the bacteria metabolize the volatile products released by sebaceous, eccrine, and apocrine glands. Therefore, differences in the individual skin microbiota can be translated into differences in the odor signature of each individual, modifying the composition and intensity of kairomones released into the air (Verhulst et al., 2010b; Smallegange et al., 2011; Ruiz-López, 2020). Moreover, individual characteristics regarding the distribution, number and activity of apocrine, eccrine and sebaceous glands influence the composition of the skin microbiota (Smallegange et al., 2011), producing a tightly interdependent cycle. Skin disorders, such as eczema, can also influence susceptibility to mosquito bites (Harford-Cross, 1993), potentially because these disorders modify the skin microbiota. Taken together, the basis of particularities in the release of skin-derived mosquito attractant may be alterations in epidermal tissue (with impacts on the skin microbiota) or on the skin microbiota *per se* (with impacts on the metabolism of glandular products).

The community of microorganisms existing on the healthy human skin varies considerably between individuals (Verhulst et al., 2010b; Oh et al., 2014). On the other hand, individual skin microbial communities remain relatively stable over time (Takken & Verhulst, 2017), as supported by robust metagenomic data (Oh et al., 2016). Together, this information strengthens the idea that the skin microbiota is, at least in part, responsible for an individual odor signature. In this sense, a number of experimental studies demonstrate the importance of skin microbiota in susceptibility to mosquitoes.

The human attractiveness to mosquitoes is altered by the diversity and abundance of the skin microbiota as shown by an *in vivo* study with *An. gambiae* (*s.s.*) mosquitoes. High abundance, but low diversity of bacteria, was associated with increased attractiveness to this mosquito species. Conversely, high microbial diversity was associated with reduced attractiveness (Verhulst et al., 2011). These data reinforce that the susceptibility to mosquitoes is not determined simply by a “high” or “low” release of volatile compounds, but also by the combination and levels of each of the kairomones blend released into the air by a particular person (Smallegange et al., 2011).

Also, the community of microorganisms existing on the human skin varies between different body parts of the same individual (Verhulst et al., 2010b; Oh et al., 2014, 2016). Volatile organic compounds produced *in vitro* by microorganisms from human feet, particularly staphylococci, corynebacteria and propionibacteria, are potent attractants of the anthropophilic *An. gambiae* (*s.s.*) mosquitoes (Verhulst et al., 2009). Even *An. arabiensis*, which generally feeds on human blood only opportunistically, can also be attracted to odors produced by human skin bacteria, as reported by Busula et al. (2017b). Interestingly, in their study, *An. arabiensis* showed increased attraction to odors from human skin bacteria compared to odors from chicken and cow bacteria (Busula et al., 2017b). In summary, the scenario presented above robustly demonstrates that differences in the composition of the skin microbiota of different individuals affect susceptibility to mosquitoes. This is especially true considering that the bacteria present on the skin metabolize substances from the skin that can become attractive to the mosquitoes.

Finally, some limitations related to the studies mentioned in this section should be considered. Skin bacteria may be undersampled or even not considered in microbiome studies due to limitations in the sampling and analysis steps. Similarly, studies of human skin odor blends based on gas chromatography/mass spectrometry can capture only certain classes of compounds. Considering these limitations, it is difficult to know which correlations between microorganisms or volatile compounds are indeed causative. As a consequence, these limitations may be reflected in associations between skin bacterial profiles and varied attractiveness to mosquitoes.

### 3.4 Diet

As reviewed by Bellani (2015), monozygotic twins under different diets exhale different odors at levels detectable by humans and trained dogs, and the consumption of meat by men modify the perception of male smell by women. Similarly, the consumption of garlic significantly changes the perception of human odor (Bellani, 2015). Taken together, these data show that diet plays a fundamental role in the variation of human body odor.

Vitamin B supplementation and garlic consumption have been suggested as mosquito repellent strategies, especially in popular media. However, in a controlled study performed by Ives & Paskewitz (2005), no effect of vitamin B supplementation on human attractiveness/repellence to *An. stephensi* was observed. To the best of our knowledge, there are no other data that consistently demonstrate any protective effect of vitamin B intake against mosquitoes. Similar to vitamin B, garlic consumption has no effect as an *Ae. aegypti* repellent, as shown in a double-blinded, placebo-controlled trial involving humans (Rajan et al., 2005).

A study performed by Shirai et al. (2002) suggested that alcohol consumption may increase the attraction of *Ae. albopictus* to humans. Subsequently, Lefèvre et al. (2010) showed that alcohol consumption also increases human attractiveness to *An. gambiae* (*s.s.*), potentially due to changes in body odor caused by alcohol metabolism. Beer was the source of alcohol in both studies mentioned above. Thus, it can be concluded that there is some evidence showing that beer consumption could be a risk factor for mosquito bites and, consequently, malaria and infection by arboviruses. It is still necessary to investigate whether the consumption of other alcoholic beverages (e.g. wine, distilled drinks) has the same effect on the attractiveness to mosquitoes. Recently, caffeine was identified on the human skin surface (Wooding et al., 2021), suggesting that the consumption of beverages (in this case coffee or derivatives) may indeed modify the human odor signature, making it possible to speculate that highly aromatic beverages and foods could affect human attractiveness to mosquitoes.

In a study performed by Paskewitz et al. (2018), the consumption of bananas (*Musa acuminata*) increased human attractiveness to *An. stephensi* and *An. gambiae*. Similar to what potentially occurs with beer, the digestion of bananas can modify human odors, also modifying human susceptibility to mosquitoes. In the same study, the consumption of green grapes (*Vitis vinifera*) showed no effect on mosquito attraction (Paskewitz et al., 2018).

In conclusion, contrary to popular belief, garlic and vitamin B, have no apparent effect as repellents (Ives & Paskewitz, 2005; Rajan et al., 2005). On the other hand, some drinks and foods, such as beer and bananas, can increase human attractiveness to mosquitoes (Shirai et al., 2002; Lefèvre et al., 2010; Paskewitz et al., 2018). Considering that human diet can modify the metabolic rate and the release of different odor-related volatile metabolites (Havlicek & Lenochova, 2006; Ajibola et al., 2013; Baranska et al., 2013; Zuniga et al., 2017), it is not surprising that some foods and alcoholic beverages can have an impact on the release of kairomones. However, as shown in this section, the literature on these aspects is quite scarce and further studies on the effects of diet on susceptibility to mosquitoes are needed. Finally, it is still necessary to know the amount and frequency of a particular food or drink that would need to be consumed in normal situations, not just under experimental conditions, for the diet to have an important effect on human susceptibility to mosquitoes.

### 3.5 Human genetics

The influence of factors strictly connected with the human biological makeup on susceptibility to mosquitoes is still poorly understood. In this context, age is potentially involved in susceptibility to mosquito bites; adults would have greater attractiveness to mosquitoes compared to children. The influence of the sex of human hosts on the preference of mosquitoes is the subject of debate (Takken & Verhulst, 2013). Women perceive themselves as more attractive to mosquitoes than men (Jones et al., 2017). However, the influences of age and sex are still not clear, mainly because they are not significant determinants of human attractiveness to mosquitoes in some situations (Qiu et al., 2006). Although it has already been suggested that blood type (ABO groups) could influence human attractiveness to mosquitoes, this association was later refuted (Lindsay et al., 1993). On the other hand, there is a growing body of evidence indicating that susceptibility to mosquitoes may have a genetic basis.

Studies with twins showed that human genetic factors are indeed involved in the susceptibility to mosquito bites (Kirk et al., 2000; Fernández-Grandon et al., 2015). This finding is quite plausible, since volatile odor molecules, as well as other human metabolites, are produced under the influence of an individual genetic background (Martin et al., 2010; Harker et al., 2014; Shin et al., 2014). For instance, the single-nucleotide polymorphism 538G/A of the *ABCC11* gene affects the production of human axillary odor (Martin et al., 2010; Harker et al., 2014), which is quite relevant to the point under discussion, once sweat production increases the attraction of mosquitoes (Khan et al., 1969). Genetic factors can influence the release patterns of individual kairomones directly through modification in the production and release of volatile odor molecules, or indirectly through changes in the composition of the individual microbiota that metabolizes skin gland products (Verhulst et al., 2010b).

It is well established that the human odor signature is partially determined by genetic factors, including human leukocyte antigen (HLA) alleles (Verhulst et al., 2010b). In a study that combined odor analysis from 48 volunteers, mosquito attractiveness testing data and HLA gene analysis (HLA-A, HLA-B, HLA-Cw, HLA-DRB and HLA-DQB typing), the HLA Cw∗07 allele was associated with increased human attractiveness to *An. gambiae* (*s.s.*) mosquitoes (Verhulst et al., 2013). Of note, lactic acid, 2-methylbutanoic acid, tetradecanoic acid and octanal were attributed to increased attractiveness. The influence of HLA alleles on the production of tetradecanoic acid (observed variance of 4.5%) may explain, at least partially, the impact of HLA on human attractiveness to *An. gambiae* (*s.s.*) mosquitoes (Verhulst et al., 2013). The small sample size is a limitation of the study by Verhulst et al. (2013), especially with respect to the assay involving HLA alleles. The impact of HLA alleles on the variability in human attractiveness to mosquitoes seems to be relevant, but it is not yet fully established in the field of study concerning human-mosquito interactions and therefore should be interpreted with caution.

More recently, other genetic factors have been associated with differences in susceptibility to mosquitoes. A genome-wide association study (GWAS) involving 16,576 European individuals (Jones et al., 2017) found that self-reported human attractiveness to mosquitoes is influenced by the following SNPs (genetic loci in parentheses): rs309403 (*BC045668* – *CETN4P*), rs1858074 (*ACSL6* – *IL-3*), rs9268659 (*HLA-DRA*), rs521977 (*SLC44A4*), rs76338894 (*AK125078* – *MIR4689*), rs3132479 (*HLA-C – MICA*), and rs139253612 (*CLMP – MIR4493*). Of these, rs309403 (4q27; BC045668 – *CETN4P*), rs1858074 (5q31.1; *ACSL6* – *IL-3*) and rs9268659 (6p21.32; *HLA-DRA*) are of special importance, showing associations with *P*-values of 6.8 × 10^−23^, 2.4 × 10^−9^ and 3.5 × 10^−9^, respectively. Corroborating previous data (Verhulst et al., 2013), genes of the immune system (especially considering HLA alleles) appear to be primarily responsible for modulating human susceptibility to mosquito bites (Jones et al., 2017). Interestingly, the same study also found a genetic basis for self-reported mosquito bite reaction size and itchiness caused by bites, with important involvement of immune system-related genes (Jones et al., 2017). The use of self-reported data is a limitation of this work and should be considered when interpreting the results mentioned above.

In addition to affecting susceptibility to mosquitoes *per se*, human genetic factors also affect individual susceptibility to pathogens transmitted by mosquitoes and the clinical characteristics of infections. Several studies focused on gene variants exemplify this aspect. For example, human homozygous carriers of the *CCR5* gene variant CCR5Δ32 (rs333) have an increased risk of symptomatic West Nile virus infection, an important emerging mosquito-borne disease in many countries (Glass et al., 2006; Ellwanger et al., 2020a). The *TNF-α-*308G/A polymorphism modulates the susceptibility to symptomatic dengue (Santos et al., 2017). The *ICAM-1* K469E polymorphism may impact the susceptibility to Japanese encephalitis virus infection (Baluni et al., 2018). Also, Toll-like receptor (TLR) genes have important influences on mosquito-borne diseases. The heterozygous genotype of *TLR2* Δ22 variant was associated with protection from cerebral *P. falciparum*-linked malaria (Greene et al., 2012). Polymorphisms of the *TLR-7* and *TLR-8* genes influence the risk of chikungunya virus infection (Dutta & Tripathi, 2017).

In addition to understanding the potential effects that specific genetic polymorphisms may have on individual susceptibility to mosquitoes, it is important to consider the interactions between different genes (e.g. epistasis, haplotype effects) as well as gene-environment interactions. These types of interactions can significantly affect (reducing or increasing) the impact of host genetics on individual susceptibility to mosquitoes (Martinez et al., 2021).

In conclusion, human genetic factors influence multiple aspects of mosquito-human interactions, including susceptibility and morbidity associated to mosquito-borne diseases. HLA alleles potentially play a determining role in individual susceptibility to mosquitoes, through its influence on the release of mosquito attractants. However, it is likely that gene polymorphism affects the production of human glandular products and skin microbiota and has an important impact on mosquito-human interactions, representing an additional research gap to be explored.

## 4 Other factors affecting human-mosquito interactions: the role of mosquito genetics, pathogens and environment

The odor-guided behavior of mosquitoes is determined by intrinsic factors of mosquitoes and extrinsic components from the environment (Hill & Ignell, 2021). Focusing on mosquito-human interactions, the behavior of blood-feeding mosquitoes depends on anthropophily (predilection to feed on humans), endophagy (preference for biting inside houses), and endophily (indoor resting behavior after a blood meal) (Campos et al., 2019). In this sense, differences in the hematophagy patterns and aggressiveness of each species significantly affect mosquito-human interactions (Consoli & Oliveira, 1994; Forattini, 2002). Similarly, the co-existence of different vectors influences the hematophagy of some species of mosquitoes on the human host. For example, mosquitoes of the subgenus *Nyssorhynchus* are capable of transmitting human malaria, but they generally act as secondary vectors, especially in the presence of *An. darlingi* (Consoli & Oliveira, 1994). As mentioned above, these behavioral and ecological components are modulated by many factors extrinsic and intrinsic to mosquitoes, including human components (e.g. the active response of each individual to the presence of the mosquito: intensely repel or not repel mosquitoes with body movements), environment (considering biotic and abiotic factors) and mosquito-linked characteristics, such as genetics.

Genetic factors of the mosquitoes have significant impacts on feeding characteristics, including host preference/selection (Kilpatrick et al., 2007; Main et al., 2016) and biting behavior (Campos et al., 2019). Some species of mosquitoes have generalist characteristics concerning blood source and, therefore, can feed on a wide variety of vertebrates (Takken & Verhulst, 2013). For instance, *An. obscurus* and *Coquillettidia* spp. have generalist host preferences (Bakker et al., 2020). Also, *An. arabiensis* and *Culex quinquefasciatus* feed on humans or other vertebrates, in similar proportions. On the other hand, some species feed on a narrow range of vertebrates, assuming a specialist behavior concerning blood-feeding. In this sense, *An. gambiae* (*s.s.*) and *Ae. aegypti* have a great preference for humans compared to other vertebrates. Although the preference of mosquitoes for certain vertebrates is greatly influenced by the availability of vertebrates in the analyzed environment, this preference is also influenced by evolutionary and genetic factors (Takken & Verhulst, 2013). Thus, from a broader perspective, it can be speculated that some species of mosquitoes or mosquito populations are more likely to identify variations in the release of human-derived kairomones, a trait that would have a genetic basis and important impacts on human susceptibility to mosquitoes. In accordance with this assumption, a recent study showed that human odor signature induces a unique neural code in the brain of *Ae. aegypti* mosquitoes, which helps these vectors differentiate humans from other animals (Zhao et al., 2020).

In addition to arthropod vector genetics, other factors associated with mosquitoes are important for the risk of pathogen transmission. The vectorial capacity (importance of a mosquito as a vector) and vector competence (one component of vectorial capacity) are crucial factors in the determination of the risk of pathogen transmission and vary between different species of mosquitoes (Kramer & Ciota, 2015). Similarly to mosquito behavior, vectorial capacity is determined by extrinsic factors (e.g. population density, mosquito-human contact, temperature, land-use changes) and intrinsic factors of each mosquito species or population (e.g. genetics, immunity, salivary and midgut barriers, tolerance to parasites, microbiome, virome) (Kramer & Ciota, 2015; Dharmarajan et al., 2019; Cansado-Utrilla et al., 2021).

A recent study (Tallon et al., 2020) showed that the dengue virus (serotype 1) mosquito infection modifies the behavior of *Ae. aegypti* since the virus can stimulate the female’s spatial exploration, besides increasing the mosquito sensitivity to kairomones. These processes are probably due to the effect of the dengue virus on the neural responsiveness of mosquito antenna to kairomones (Tallon et al., 2020). Also, other authors reported that dengue infection can increase (up to ∼50%) the locomotor activity of *Ae. aegypti* (Lima-Camara et al., 2011).

In a study performed by Vogels et al. (2017), West Nile virus infection decreased the host-seeking response of *Culex pipiens* mosquitoes, potentially due to virus alterations in the central nervous system of the mosquito. According to Jackson et al. (2012), the blood-feeding behavior of *Ae. triseriatus* and *Ae. albopictus* is altered by La Crosse virus infection. Looking specifically at blood-meal size, infected mosquitoes took less blood compared to uninfected mosquitoes (Jackson et al., 2012). Reduced blood-meal size can result in more frequent feedings, increasing the chances of virus transmission. In this sense, La Crosse virus infection could cause enhanced transmission and increased vectorial capacity of *Ae. triseriatus* (Yang et al., 2019). The work performed by Yang et al. (2019) suggested that La Crosse virus-induced reduction of serotonin levels may be involved in blood-feeding alterations in *Ae. triseriatus* mosquitoes.

A recent systematic review and meta-analysis considering different species of mosquito and pathogens concluded that infected mosquitoes have altered behavior to repellents. In general, repellents were less effective against infected mosquitoes (Lajeunesse et al., 2020). Taken together, in addition to manipulation of the release patterns of kairomones by vertebrate hosts (Cordy, 2020; Cozzarolo et al., 2020; or see the case of malaria in Section 3.2 of this review), the studies mentioned above indicate that mosquito-associated pathogens can also alter the sensitivity of mosquitoes to these molecules, as well as mosquito behavior.

Depending on the conditions evaluated, human components, environmental factors and mosquito-related characteristics will have a greater or lesser influence on the chances of human suffering from mosquito bites. The availability of hosts can increase or decrease the influence of mosquito genetic factors acting on host preference (Kilpatrick et al., 2007). For example, an anthropophilic mosquito can feed on a non-human vertebrate if it is the only source of food available (Takken & Verhulst, 2013). The presence of livestock in the household can significantly alter the resting and feeding behaviors of malaria vectors (Mayagaya et al., 2015), which likely modifies the risk of human *Plasmodium* infection. The feeding of *An. arabiensis* and *An. funestus* (*s.l.*) on a human host is much reduced (up to ∼50%) in the presence of cattle at household, as observed in a study performed in Tanzania (Mayagaya et al., 2015). Conversely, the opposite may also be true. A non-anthropophilic mosquito may feed on a human if it is the only source of blood. In this case, it is possible that environmental factors (availability of vertebrates) overlap with mosquito genetic factors, displacing or even supplanting at least momentarily, the natural preferences. However, this assumption needs to be confirmed experimentally. Similarly, the effect of human genetics and its influence on the release of kairomones may have greater or lesser importance on susceptibility to mosquitoes depending on the number and diversity of humans susceptible to bites (availability of blood) in a given environment.

**Fig. 3:**
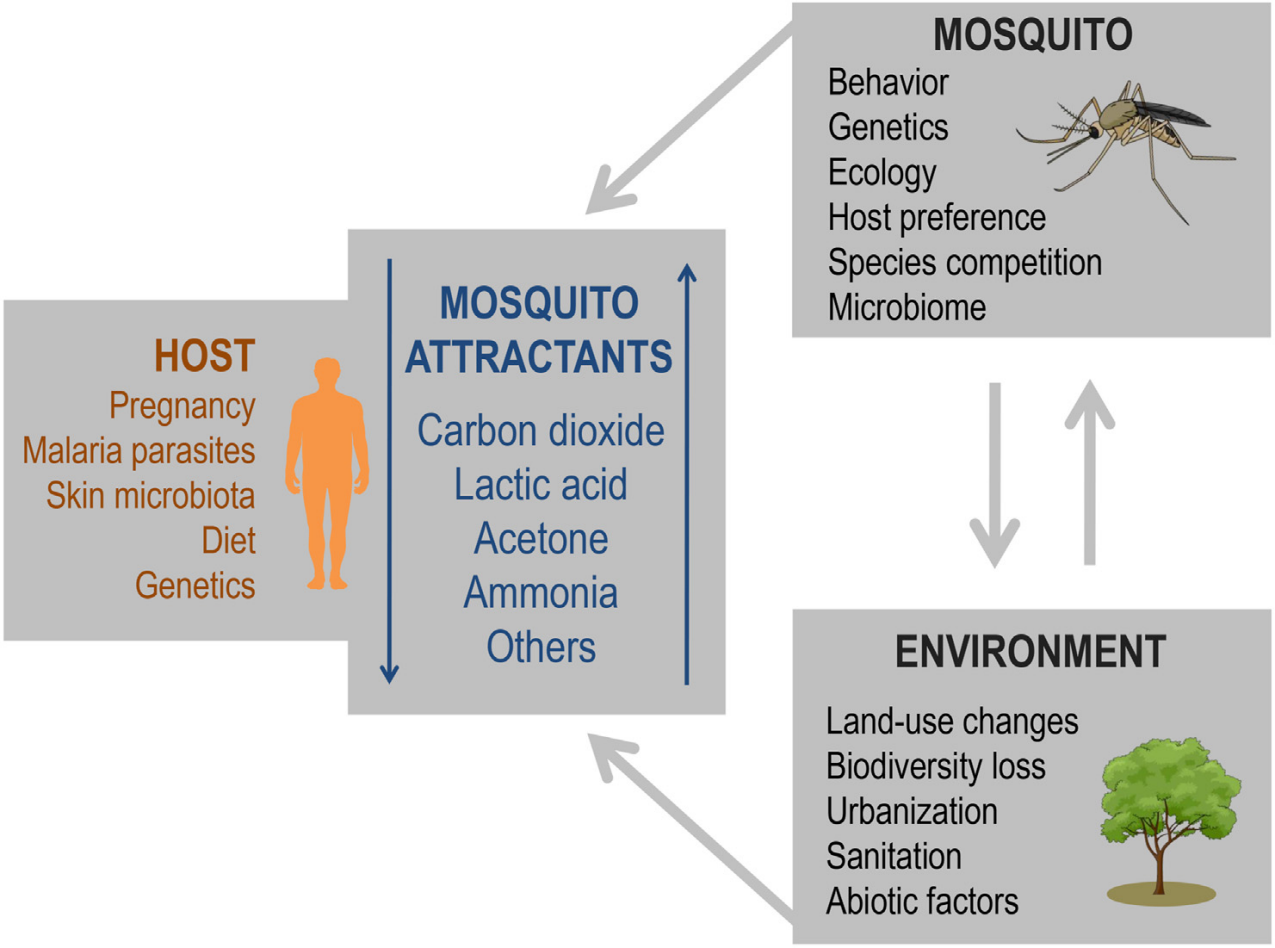
Factors that interfere with human-mosquito interactions. Skin microbiota, pregnancy, malaria parasites (*Plasmodium* infection), human genetic factors (e.g. HLA alleles) and diet affect the release patterns of mosquito attractants (kairomones), impacting human-mosquito interactions. In association with mosquito-linked characteristics (e.g. mosquito genetics, infectious status) and environmental components (e.g. wind, humidity), these factors can increase or decrease the susceptibility of an individual to mosquito bites, affecting the risk of infection by pathogens transmitted by mosquitoes (arboviruses, malaria parasites, among others). These factors can affect either the intensity of production or the composition of mosquito attractants. See the main text for references. Human illustration was obtained from *Servier Medical Art* (available at https://smart.servier.com, under a Creative Commons Attribution 3.0 Unported License). Mosquito and tree illustrations were obtained from *Mind the Graph* (available at www.mindthegraph.com).

Figure 3 summarizes the factors that interfere with human-mosquito interactions. The impact of the environment on human-mosquito interactions is increasingly evident. Deforestation, land use and climate change have a major impact on the distribution of mosquito populations. In general, these factors facilitate the proliferation of these insects and increase the risks of mosquito-borne diseases (Ellwanger et al., 2020b). Deforestation can favor the proliferation of mosquitoes that transmit diseases over mosquito species without medical importance, once environments modified by anthropogenic activities will be more easily occupied by species adapted to human-related landscapes, such as *Ae. aegypti* (Franklinos et al., 2019). Anthropogenic changes in the natural environment can also drive evolutionary aspects of mosquitoes. According to a recent study by Rose et al. (2020), long and dry seasons, urbanization and the resulting intimate relationship between humans and *Ae. aegypti* in these conditions, such as the mosquito use of human-stored water sites, contributed to the selection of the anthropophilic characteristic observed in this mosquito species. As a consequence of these human-mosquito interactions, this anthropophilic characteristic was fixed in the genome of *Ae. aegypti* populations found especially in dry and densely populated places (Rose et al., 2020). These results clearly exemplify how anthropogenic changes in the environment can result in biological changes in mosquito populations with impact on public health, reinforcing the need for mosquito control strategies. Of note, vector control strategies should integrate mechanisms to reduce mosquito populations especially by improving the sanitary conditions and health of the environment, particularly in densely populated areas (WHO, 2017; Ellwanger et al., 2021).

Finally, the role of wind and air flows on human-mosquito interactions must be highlighted. The wind modulates the population susceptibility to mosquito-borne diseases because this abiotic factor determines the chances of mosquitoes finding human-derived kairomones in the environment, once the distribution of the volatile compounds is influenced by wind direction and intensity (Cardé & Willis, 2008; Cummins et al., 2012; Ellwanger & Chies, 2018). Of note, the host-derived volatile compounds can disperse in the environment as “puffs” or “packets” of odors, under the influence of the wind direction, and not necessarily through gradual molecular diffusion from the host towards the insect (Cardé & Willis, 2008). The effect of the wind was especially evidenced by the chances of *Anopheles* mosquitoes interacting with human-derived kairomones, which have a substantial impact on the risk of malaria transmission (Endo & Eltahir, 2018). Furthermore, the wind can contribute to the dispersion, or wind-mediated transport, of mosquitoes over long distances (Ritchie & Rochester, 2001; Huestis et al., 2019), increasing the susceptibility of populations to mosquito bites and mosquito-borne diseases even in areas with limited mosquito breeding sites.

Other abiotic factors besides wind can have a major effect on mosquito populations, human-mosquito interactions, and consequently mosquito-borne diseases. For example, the levels of humidity, temperature, rain patterns and air pollution affect the dynamic of dengue virus transmission in Brazil (Rosa-Freitas et al., 2006; Câmara et al., 2009; Viana & Ignotti, 2013; Carneiro et al., 2017). It is therefore essential to consider that the variability in human attractiveness to mosquitoes will be affected by circumstantial influences associated with the abiotic factors of a given environment.

## 5 Conclusion and perspectives

Human susceptibility to mosquitoes is influenced by environmental components, mosquito-linked factors and human-related characteristics and conditions. Among the latter, the most well-characterized to date are pregnancy, *Plasmodium* infection, skin microbiota, genetics, and potentially diet. The importance of each of these factors will be greater or lesser, depending on the environment, availability of vertebrates and species of mosquito. In general, some patterns are evident: pregnancy and *Plasmodium* infection increase human attractiveness to mosquitoes, especially to *Anopheles* species; the diet appears to have some influence on the release of human-derived kairomones, although this topic still needs further investigation; skin microbiota and human genetics, especially HLA alleles, modulate the production of kairomones, influencing the susceptibility to mosquitoes. These influences are summarized in Fig. 3. The patterns reviewed in this article are important for studies focused on infectious disease dynamics and, especially, for the development of better mosquito control strategies (e.g. development of better repellents for individual use). Considering the public health perspective, the variability in human susceptibility to mosquitoes may impact the risk of infection by mosquito-borne diseases in endemic areas. This variability must be considered in mosquito control strategies.

## CRediT author statement

Joel Henrique Ellwanger: conceptualization, investigation, writing - original draft, writing - review and editing. Jáder da Cruz Cardoso: conceptualization, investigation, writing - review and editing. José Artur Bogo Chies: investigation, writing - review and editing, supervision. All authors read and approved the final manuscript.

## Funding

Joel Henrique Ellwanger receives a postdoctoral fellowship from Coordenação de Aperfeiçoamento de Pessoal de Nível Superior (Programa Nacional de Pós-Doutorado/CAPES, Brazil). José Artur Bogo Chies receives a research fellowship from Conselho Nacional de Desenvolvimento Científico e Tecnológico (Bolsista de Produtividade em Pesquisa 1A/CNPq, Brazil) and has a research project funded by 10.13039/501100002322CAPES (Brazil).

## Declaration of competing interests

The authors declare that they have no known competing financial interests or personal relationships that could have appeared to influence the work reported in this paper.
